# Heat Shock Protein 70 Family Members Interact with Crimean-Congo Hemorrhagic Fever Virus and Hazara Virus Nucleocapsid Proteins and Perform a Functional Role in the Nairovirus Replication Cycle

**DOI:** 10.1128/JVI.00661-16

**Published:** 2016-09-29

**Authors:** Rebecca Surtees, Stuart D. Dowall, Amelia Shaw, Stuart Armstrong, Roger Hewson, Miles W. Carroll, Jamel Mankouri, Thomas A. Edwards, Julian A. Hiscox, John N. Barr

**Affiliations:** a Virology & Pathogenesis, Public Health England, Porton Down, Salisbury, United Kingdom; b School of Molecular and Cellular Biology, Faculty of Biological Sciences, and Astbury Centre for Molecular and Structural Biology, University of Leeds, Leeds, United Kingdom; c Institute of Infection and Global Health, University of Liverpool, Liverpool, United Kingdom; d NIHR Health Protection Research Unit in Emerging and Zoonotic Infection, Liverpool, United Kingdom; Wake Forest University

## Abstract

The Nairovirus genus of the Bunyaviridae family contains serious human and animal pathogens classified within multiple serogroups and species. Of these serogroups, the Crimean-Congo hemorrhagic fever virus (CCHFV) serogroup comprises sole members CCHFV and Hazara virus (HAZV). CCHFV is an emerging zoonotic virus that causes often-fatal hemorrhagic fever in infected humans for which preventative or therapeutic strategies are not available. In contrast, HAZV is nonpathogenic to humans and thus represents an excellent model to study aspects of CCHFV biology under conditions of more-accessible biological containment. The three RNA segments that form the nairovirus genome are encapsidated by the viral nucleocapsid protein (N) to form ribonucleoprotein (RNP) complexes that are substrates for RNA synthesis and packaging into virus particles. We used quantitative proteomics to identify cellular interaction partners of CCHFV N and identified robust interactions with cellular chaperones. These interactions were validated using immunological methods, and the specific interaction between native CCHFV N and cellular chaperones of the HSP70 family was confirmed during live CCHFV infection. Using infectious HAZV, we showed for the first time that the nairovirus N-HSP70 association was maintained within both infected cells and virus particles, where N is assembled as RNPs. Reduction of active HSP70 levels in cells by the use of small-molecule inhibitors significantly reduced HAZV titers, and a model for chaperone function in the context of high genetic variability is proposed. These results suggest that chaperones of the HSP70 family are required for nairovirus replication and thus represent a genetically stable cellular therapeutic target for preventing nairovirus-mediated disease.

**IMPORTANCE** Nairoviruses compose a group of human and animal viruses that are transmitted by ticks and associated with serious or fatal disease. One member is Crimean-Congo hemorrhagic fever virus (CCHFV), which is responsible for fatal human disease and is recognized as an emerging threat within Europe in response to climate change. No preventative or therapeutic strategies against nairovirus-mediated disease are currently available. Here we show that the N protein of CCHFV and the related Hazara virus interact with a cellular protein, HSP70, during both the intracellular and extracellular stages of the virus life cycle. The use of inhibitors that block HSP70 function reduces virus titers by up to 1,000-fold, suggesting that this interaction is important within the context of the nairovirus life cycle and may represent a potent target for antinairovirus therapies against which the virus cannot easily develop resistance.

## INTRODUCTION

The Bunyaviridae family of trisegmented negative-sense RNA viruses comprises five genera, namely, Orthobunyavirus, Hantavirus, Nairovirus, Phlebovirus, and Tospovirus (1). The Nairovirus genus contains several serogroups, one of which is the Crimean-Congo hemorrhagic fever virus (CCHFV) serogroup, with sole members CCHFV and the genetically distinct Hazara virus (HAZV) (2) that are formally grouped under the same species name of CCHFV. CCHFV is a risk group 4 human pathogen, responsible for a devastating disease for which preventative or therapeutic measures do not exist (3). Transmission of CCHFV to humans often occurs by the bite of infected ixodid ticks of the Hyalomma genus (4), and the human case-fatality rate can exceed 60% (5). In recent years, the occurrence of CCHFV-mediated disease has been newly reported in many Mediterranean countries (3), likely as a consequence of the increasingly broad habitat and population size of its tick vector, with the increases possibly occurring in response to climate change (6). CCHFV is now recognized as a potential threat to human health in the densely populated regions of Northern Europe (7). In contrast, HAZV has not been associated with serious human disease and is classified as a risk group 2 pathogen. HAZV infection of type 1 interferon receptor-deficient mice shares clinical features of CCHFV-mediated disease in humans and represents an accessible CCHFV infection model (8). Taken together, these findings suggest that HAZV is a useful surrogate that can be used to study the molecular, cellular, and disease biology of the highly pathogenic CCHFV, as well as of other nairoviruses responsible for serious human and animal diseases. Such nairoviruses include Erve virus, which causes “thunderclap” headaches in humans (9), and Nairobi sheep disease virus, which is responsible for hemorrhagic gastroenteritis in livestock such as sheep and goats (10).

The nairovirus genome comprises three strands of negative-sense RNA that are named small (S), medium (M), and large (L), reflecting their relative sizes. The S segment encodes the nucleocapsid (N) protein, the M segment encodes the envelope glycoproteins Gn and Gc, and the L segment encodes the viral RNA-dependent RNA polymerase (RdRp). As with all bunyaviruses, the nairovirus genome and antigenome are encapsidated by multiple copies of the viral N protein to form a ribonucleoprotein (RNP) complex. This N-RNA association is thought to be critical for the virus replication cycle, and only in the form of the RNP can the CCHFV genome be transcribed and replicated (11). The formation of the nairovirus RNP is also dependent on the ability of the N protein to interact with itself to form extended oligomers, and details of this interaction have been revealed at the atomic level by the CCHFV N protein crystal structure, reported from several groups, including us (11–13). More recently, the solution of the HAZV N protein crystal structure by others and ourselves (14, 15) reveals a high degree of structural similarity with CCHFV N, providing further support for the use of HAZV as a model for CCHFV, in particular for functions related to their N proteins.

In addition to these interactions with viral RNP components, bunyavirus N proteins are known to interact with multiple cellular proteins, resulting in changes to a range of cellular functions such as disruption of host cell signaling pathways (16, 17), cellular mRNA cap binding (18, 19), and the recruitment of host cell translation machinery to enhance viral protein expression (20, 21). In comparison to the information available regarding bunyaviruses from other genera, less is known about the cellular interacting partners specific to the nairovirus N protein. Two interacting cellular proteins have previously been identified, namely, actin and the large GTPase MxA (22, 23). Although the functional consequences of these interactions within the virus replication cycle have not been fully elucidated, those studies showed that N was capable of interacting with cellular proteins.

Identification of interactions between host and virus components is important as it holds the potential to result in development of novel disease prevention strategies that focus on either host or viral targets. Therapeutic strategies that target host cell components possibly reduce the likelihood of resistance developing due to the relatively low rate of cellular DNA genetic change compared to the more rapid virus evolution. This is especially pertinent in the case of RNA viruses such as CCHFV, which have high mutation rates due to their error-prone RNA polymerases (24).

In order to better understand the interaction between the nairovirus N protein and the host cell, we performed a mass spectrometry-based analysis of its coprecipitating cellular proteins. Independent validation by immunological means revealed that cellular chaperones, particularly those of the HSP70 family, were abundant interactors. We showed that this interaction was maintained throughout both the intracellular and extracellular stages of the nairovirus replication cycle and that ablation of HSP70 activity using small-molecule inhibitors resulted in a 1,000-fold reduction of infectious HAZV production. This suggests that HSP70 family members perform an important role within the nairovirus replication cycle and that inhibition of HSP70 function may be an important therapeutic strategy for currently untreatable viral diseases.

## MATERIALS AND METHODS

### Mammalian cell culture.

SW13 cells, which are of human adrenal cortex carcinoma cell origin (25), were a kind gift from Stuart Dowall and Roger Hewson of Public Health England (PHE). HEK293T cells, which are of human embryonic kidney cell origin (26), and A549 cells, which are of human alveolar carcinoma cell origin (27), were obtained from the Health Protection Agency Culture Collections (HPACC). All cell lines were maintained in Dulbecco's modified Eagle's medium (DMEM; Sigma-Aldrich) supplemented with 10% fetal bovine serum (FBS; Invitrogen), 100 IU of penicillin/ml, and 100 μg streptomycin/ml and incubated at 37°C in the presence of 5% CO_2_. To perform stable isotope labeling of amino acids in cell culture (SILAC) differential cell labeling studies, HEK293T cells were grown in SILAC DMEM supplemented with 10% dialyzed FBS (Dundee Cell Products), 100 IU of penicillin/ml, and 100 μg streptomycin/ml. Media were named either “light” (R0K0) or “medium” (R6K4), depending on the inclusion of stable isotopes of carbon (^13^C) or hydrogen (^2^H) in arginine and lysine amino acids. Cells were grown in these media for a minimum period of 2 weeks (>7 cell divisions) prior to transfection to ensure >95% cellular protein labeling. For both light-medium- and medium-medium-labeled cells, four 10-cm^2^ dishes were seeded with 1.25 × 10^6^ HEK293T cells each 24 h prior to transfection.

### Plasmids encoding nairovirus N proteins.

The CCHFV N protein cDNA sequence from strain Baghdad-12 was cloned into the pEGFP-C2 vector (Clontech) to allow mammalian cell expression of the CCHFV N protein fused to the C terminus of enhanced green fluorescent protein (EGFP), and the resulting plasmid (pEGFP-CCHFV-N) was verified by sequencing. Expression of native CCHFV N was performed by cloning the cDNA described above into pCAGGS to derive plasmid pCAGGS-CCHFV-N. Expression of both the EGFP-N fusion protein (EGFP–CCHFV-N) and the native CCHFV N protein was confirmed by immunofluorescence (IF) and confocal microscopy as well as by Western blot analysis using either an anti-EGFP antibody (Santa Cruz Biotechnology; sc8334) or an anti-CCHFV N antibody generated in-house (described below).

### Bacterial cell expression of nairovirus N proteins and antibody production.

cDNAs comprising the open reading frames of the N protein of CCHFV strain Baghdad-12 (GenBank accession number AJ538196.1) and HAZV strain JC280 (GenBank accession number M86624.1) optimized for bacterial expression were synthesized by Dundee Cell Products. Expression of CCHFV N and HAZV N in Escherichia coli was carried out as described previously (Carter et al. [11]; Surtees et al. [14]). Following purification, N purity was confirmed to be >95% by SDS-PAGE, and 1 mg N protein in a 1-ml volume was used for antibody production in sheep by AltaBiosciences Ltd. (United Kingdom).

### Transfection.

HEK293T cells were transfected using Ca_3_(PO_4_)_2_ with 10 μg plasmid DNA encoding EGFP–CCHFV-N or EGFP per 10-cm^2^ dish. For immunoprecipitation (IP), four 10-cm^2^ dishes were transfected. Plasmid DNA was added to a solution containing sterile filtered 244 mM CaCl_2_, which was added to sterile 2× HBS (274 mM NaCl, 302 μM Na_2_HPO_4_·12H_2_O, 55 mM HEPES [pH 7]). Solutions of CaCl_2_, DNA, and 2× HBS were incubated at room temperature for 30 min, prior to addition to HEK293T cells. Complete media containing transfection mixes were incubated with HEK293T cells overnight, and then the media were replaced with fresh media or cells were harvested. To visualize CCHFV N protein using indirect immunofluorescence microscopy, HUH-7 cells were transfected in 60% to 70% confluent 12-well plates using 0.7 μg pCAGGS-CCHFV-N in 100 μl Opti-MEM with 3 μl Lipofectamine 2000 (Invitrogen).

### Nairovirus infections.

CCHFV (strain Baghdad-12) was used to infect SW13 cells at a multiplicity of infection (MOI) of 1.0 under maximum containment conditions at Public Health England, Porton Down, Salisbury, United Kingdom. At 24 h postinfection, cells were lysed and lysates were used in IPs using protein G-coupled Dynabeads, as described below. For HAZV purification, strain JC280 was propagated in SW13 cells at an MOI of 0.001 under biosafety level 2 (BSL2) conditions. After 4 days, the supernatant containing infectious HAZV was harvested and clarified by centrifugation at 4,000 × *g* at 4°C for 20 min. For HAZV N IPs, subconfluent SW13 cells grown in 10-cm^2^ dishes were infected with HAZV at an MOI of 1, and 24 h postinfection, cell lysates were used in HAZV N IPs as described below.

### HAZV purification.

Clarified supernatant from HAZV-infected SW13 cells was mixed with 50% (wt/vol) polyethylene glycol (PEG) 6000–TNE buffer (0.01 M Tris [pH 7.4], 0.1 M NaCl, 1 mM EDTA) to give a final PEG 6000 concentration of 10% (wt/vol). HAZV was precipitated overnight at 4°C and then recovered by centrifugation at 4,000 × *g* for 30 min at 4°C. The resulting pellet was resuspended in 1 ml of TNE buffer and then layered on top of a continuous 5% to 25% iodixanol gradient in a PolyClear centrifuge tube (Seton) (13 by 51 mm). After ultracentrifugation at 250,000 × *g* for 2.5 h at 4°C, HAZV particles were isolated using a syringe, mixed gently, and frozen at −80°C.

### Plaque assays.

Plaque assays were performed in 12-well plates using confluent SW13 cells. After adsorption, virus was removed and replaced with media diluted 1:1 with 1.6% (wt/vol) high-viscosity carboxy-methyl cellulose (Sigma-Aldrich). At 6 days, cells were fixed in 20% (vol/vol) formalin for 1 h, washed 3 times in double-distilled water (ddH_2_O), and then stained with crystal violet to visualize plaques.

### Preparation of cell lysates.

Cells were harvested from culture dishes by scraping into media and then recovered by centrifugation at 4°C at 4,000 × *g* for 5 min, washed 3 times in phosphate-buffered saline (PBS), and then resuspended in lysis buffer (10 mM Tris-HCl [pH 7.5], 150 mM NaCl, 0.5 mM EDTA, 0.5% NP-40, 1× EDTA-free complete protease inhibitor; Roche) for IP or in radioimmunoprecipitation assay (RIPA) buffer (50 mM Tris-HCl [pH 7.5], 150 mM NaCl, 1% [vol/vol] NP-40 alternative, 0.5% [wt/vol] sodium deoxycholate, 0.1% 1× EDTA-free complete protease inhibitor cocktail) for Western blot analysis. Cell pellets were incubated in RIPA buffer for 30 min on ice, and then lysates were clarified by centrifugation at 13,000 × *g* at 4°C for 10 min and the supernatant was either used directly or stored at −20°C.

### EGFP-N immunoprecipitations (GFP-trap).

EGFP and EGFP–CCHFV-N protein IP experiments were performed using a single-domain anti-GFP antibody conjugated to agarose beads (GFP-trap; Chromotek). IPs with similarly conjugated red fluorescent protein (RFP-trap) were performed as nonbinding negative controls. Cell pellets were incubated for 30 min with 200 μl lysis buffer (10 mM Tris-HCl [pH 7.5], 150 mM NaCl, 0.5 mM EDTA, 0.5% NP-40, 1× EDTA-free protease inhibitor [Roche]). The lysate was clarified and diluted 5-fold with dilution buffer (10 mM Tris-HCl [pH 7.5], 150 mM NaCl, 0.5 mM EDTA, 1× EDTA-free protease inhibitor [Roche]). The GFP-trap/RFP-trap beads were incubated with diluted cell lysate for 2 h at 4°C and then centrifuged at 2,700 × *g* for 2 min followed by two washes with wash buffer (10 mM Tris HCl [pH 7.5], 250 mM NaCl, 0.5 mM EDTA, 1× EDTA-free protease inhibitor [Roche]) and then one wash with wash buffer containing 275 mM NaCl. After centrifugation at 2,700 × *g*, pelleted beads were resuspended in 2× LDS-sample buffer (10 mM Tris-HCl [pH 7.5], 150 mM NaCl, 0.5 mM EDTA, 1× protease inhibitor cocktail, 1× LDS, 50 mM dithiothreitol [DTT]) and heated at 95°C for 10 min. Equal volumes of labeled IP samples were mixed and analyzed by liquid chromatography-tandem mass spectrometry (LC-MS/MS).

### Native nairovirus N protein immunoprecipitation.

Recombinant protein G-coupled Dynabeads (Novex) were used to IP native nairovirus N proteins from virus-infected and transfected cell lysates using the corresponding anti-N antibodies. Dynabead IPs were carried out using a Dynabead protein G IP kit (Life Technologies) according to the manufacturer's instructions. Briefly, 50 μl Dynabeads was bound to CCHFV or HAZV N antibodies and then incubated with infected or transfected cell lysates for 15 min at room temperature. Antigen-antibody complexes were washed in wash buffer, and proteins were eluted by heating at 95°C for 10 min in 2× LDS buffer.

### Immunofluorescence (IF).

To visualize various proteins, cells were grown on 19-mm-diameter glass coverslips housed in 12-well plates. After infection or transfection, cells were washed twice in PBS prior to fixation using either 4% formaldehyde (BHD Chemicals)–PBS for 10 min at room temperature or 4% paraformaldehyde (PFA; BHD Chemicals) diluted in PBS for 20 min at room temperature. After fixative removal, cells were washed 3 times with PBS and then permeabilized in 0.5% (vol/vol) Triton X-100–PBS for 12 min at room temperature. Permeabilized cells were washed 3 times in PBS and then blocked in 2% (vol/vol) FBS–PBS for 30 min. Primary and secondary antibody incubations were performed with antibodies diluted to the appropriate concentration in 2% (vol/vol) FBS–PBS; after blocking, cells were incubated with 50 μl of the appropriate primary antibody for 1 h at room temperature and then washed 3 times in PBS and incubated with 50 μl of the appropriate fluorescently labeled secondary antibody for 1 h at room temperature. Cells were washed in PBS prior to mounting onto glass slides using ProLong Gold antifade reagent (Life Technologies) containing DAPI (4′,6-diamidino-2-phenylindole). Slides harboring fixed and stained cells were analyzed by confocal microscopy using either an inverted laser scanning microscope (LSM; 510 Meta Axiovert 200M) or an upright LSM (510 Meta Axioplan) (Carl Zeiss Ltd.).

### MS analysis of immunoprecipitated EGFP–CCHFV-N cell lysates.

Protein samples generated by GFP-trap IPs were analyzed by SDS-PAGE and separated proteins isolated in 10 slices and subjected to in-gel digestion with trypsin. The resulting peptides were separated using an Ultimate U3000 nanoflow LC system (Dionex Corporation) consisting of a solvent degasser, micro- and nanoflow pumps, a flow control module, a UV detector, and a thermostated autosampler. A sample volume of 10 μl (comprising 2 μg) was loaded with a constant flow of 20 μl/min onto a PepMap C_18_ trap column (Dionex Corporation) (0.3 mm by 5 mm). After trap enrichment, peptides were eluted onto a PepMap C_18_ nano column (Dionex Corporation) (75 μm by 15 cm) with a linear gradient of 5% to 35% solvent B (90% acetonitrile with 0.1% formic acid) over 65 min at a constant flow rate of 300 nl/min. The high-performance LC (HPLC) system was coupled to a LTQ Orbitrap XL system (Thermo Fisher Scientific Inc.) via a nano electrospray (ES) ion source (Proxeon Biosystems). The spray voltage was set to 1.2 kV, and the temperature of the heated capillary was set to 200°C. Full-scan MS survey spectra (*m/z* 335 to 1,800) in profile mode were acquired in the Orbitrap system with a resolution of 60,000 after accumulation of 500,000 ions. The five most intense peptide ions with regard to detected abundances from the Orbitrap preview scan were fragmented by collision-induced dissociation (normalized collision energy, 35%; activation Q, 0.250; activation time, 30 ms) in the LTQ Orbitrap XL system after accumulation of 10,000 ions. Maximal filling times were 1,000 ms for the full scans and 150 ms for the MS/MS scans. Precursor ion charge state screening was enabled, and all unassigned charge states as well as singly charged species were rejected. The dynamic exclusion list was restricted to a maximum of 500 entries, with a maximum retention period of 90 s and a relative mass window of 10 ppm. The lock mass option was enabled for survey scans to improve mass accuracy. The data were acquired using Xcalibur software. LC-MS/MS analysis was performed twice on the same protein sample.

### Peptide quantification.

Quantification was performed with MaxQuant version 1.0.7.4 (28) based on the two-dimensional centroid of the isotope clusters within each SILAC pair. The generation of values corresponding to peak list, SILAC-based, and extracted ion current-based quantitation, calculated posterior error probability, false-discovery rate based on search engine results, peptide-to-protein group assembly, and data filtration and presentation was carried out using MaxQuant. The derived peak list was searched with the Mascot search engine (Matrix Science, London, United Kingdom) against a concatenated database from the International Protein Index (IPI) human protein database and the reversed sequences of all proteins. Combined data sets from the two different experiments were deposited in the PRoteomics IDEntifications database (PRIDE) using the PRIDE converter tool and are presented as an annotated table (see Data set S1 in the supplemental material).

### Western blot analysis.

Proteins were resolved on 12% SDS polyacrylamide gels and transferred to polyvinylidene difluoride (PVDF) membranes (Immobilon-P; Millipore) or fluorescence-compatible PVDF membranes (FL-PVDF) (Immobilon-FL Transfer membranes; Millipore) using a Trans-Blot semidry cell (Bio-Rad) in Towbin buffer (25 mM Tris, 192 mM glycine, 20% [vol/vol] methanol) for 1 h at 15 V. Membranes were blocked for 1 h at room temperature in 10% (wt/vol) bovine serum albumin (BSA) (Sigma-Aldrich)–Tris-buffered saline (TBS) supplemented with 0.01% Tween 20 (TBS-T) (50 mM Tris-HCl [pH 7.5], 150 mM NaCl, 0.1% Tween 20). FL-PVDF membranes were blocked for 1 h at room temperature in 50% (vol/vol) Odyssey blocking buffer (LiCor)–TBS (50 mM Tris-HCl [pH 7.5], 150 mM NaCl). Membranes were incubated with primary antibody diluted appropriately in 5% (wt/vol) BSA–1× TSB-T or in 50% Odyssey blocking buffer–TBS either for 1 h at room temperature or at 4°C overnight. Primary antibodies used include those raised against GFP (Santa Cruz Biotechnology; sc-8334), HSP70/HSC70 (Abcam; Ab6535), vimentin (Santa Cruz Biotechnology; sc-966), actin (Sigma-Aldrich; A3853), B23.1 (Santa Cruz Biotechnology; sc47725), p53 (Cell Signaling; 9282), and GAPDH (glyceraldehyde-3-phosphate dehydrogenase) (Abcam; ab8245). PVDF membranes were incubated with an appropriate secondary antibody conjugated to horseradish peroxidase (HRP) as described above. FL-PVDF membranes were similarly processed but were incubated with the appropriate fluorescently labeled secondary antibody. PVDF membranes were washed in TBS-T and once in water, and proteins were visualized using enhanced chemiluminescence on photographic film. FL-PVDF membranes were washed in TBS and once in water and then imaged using a LiCor Odyssey Sa system (LiCor).

### MS analysis of purified virus; sample preparation and tryptic digestion.

Purified virus comprising approximately 11 μg protein was denatured by heating for 10 min at 95°C in the presence of DTT. Viral proteins and controls were run approximately 1 cm into a 4% to 12% Tris-Bis gradient gel, and the proteins were visualized using Coomassie staining. The entire gel lane was excised and cut into smaller pieces, destained in 50% acetonitrile–50 mM ammonium bicarbonate (pH ∼8), reduced for 30 min at 37°C with 10 mM dithiothreitol (Sigma-Aldrich)–50 mM ammonium bicarbonate, and alkylated with 55 mM iodoacetamide (Sigma)–50 mM ammonium bicarbonate for 30 min at room temperature. Gel pieces were washed for 15 min in 50 mM ammonium bicarbonate and dehydrated with 100% acetonitrile. Acetonitrile was removed, and the gel plugs were rehydrated with 0.01 μg/μl proteomics-grade trypsin (Sigma-Aldrich) in 50 mM ammonium bicarbonate. Digestion was performed overnight at 37°C. Peptides were extracted from the gel plugs using successive 15-min incubations and 3% (vol/vol) acetonitrile–0.1% (vol/vol) trifluoroacetic acid (TFA). Peptide extracts were pooled and reduced to dryness using a centrifugal evaporator (Microfuge concentrator plus) and resuspended in 3% (vol/vol) acetonitrile–0.1% (vol/vol) TFA for analysis by mass spectrometry.

### Mass spectrometry of purified HAZV proteins.

Peptide mixtures (2 μl) were analyzed by on-line nanoflow liquid chromatography using an nanoACQUITY-nLC system (Waters MS Technologies) coupled to an LTQ-Orbitrap Velos (Thermo Fisher Scientific) mass spectrometer equipped with a nanospray ion source. The analytical column (nanoACQUITY ultraperformance LC [UPLC] BEH130 C_18_) (15 cm by 75 μm, 1.7 μm capillary column) was maintained at 35°C with a flow rate of 300 nl/min. The gradient consisted of 3% to 40% acetonitrile–0.1% formic acid for 150 min followed by a ramp of 40% to 85% acetonitrile–0.1% formic acid for 5 min. Full-scan MS spectra (*m/z* range, 300 to 2,000) were acquired by the Orbitrap at a resolution of 30,000. The top 20 most intense ions from the MS1 scan (full MS) were selected for tandem MS by collision-induced dissociation, and all product spectra were acquired in the LTQ ion trap. Spectral data were transformed to .mgf files with Proteowizard (version 3.0) (29) and used for peptide identification performed with the Mascot (version 2.3.02; Matrix Science) search engine. Tandem MS data were searched against the predicted HAZV and human proteomes (UniProt release 2014_02). Mascot search parameters were as follows: precursor mass tolerance set to 10 ppm and fragment mass tolerance set to 0.8 Da. A maximum of one missed tryptic cleavage was permitted. Carbamidomethylation (cysteine) was set as a fixed modification and oxidation (methionine) as a variable modification. Mascot search results were further processed using Percolator. The false-discovery rate was <1%. Individual ion scores of >13 indicated identity or extensive homology (*P* < 0.05). Approximate label-free spectrum-counting-based quantitation of the proteins was determined using the exponentially modified protein abundance index (emPAI) calculated automatically by the Mascot search engine (30).

### HSP70/HSC70 inhibition.

Small-molecule inhibitors VER155008 (VER) and pifithrin-μ (PIF) (Sigma-Aldrich) were both dissolved in dimethyl sulfoxide (DMSO) (Sigma-Aldrich) to make 10 mM stocks, which were further diluted in DMEM supplemented with 2% FBS to the appropriate working concentrations. Assay results were compared to those obtained with DMEM supplemented with 2% FBS containing 0.4% DMSO. A549 cells were seeded in 12-well plates such that the cells were 60% to 70% confluent when infected and were then exposed to the appropriate concentration of PIF and VER. After 1 h, cells were infected with HAZV at an MOI of 1 and subsequently replaced with fresh media containing appropriate concentrations of PIF and VER. At 24 h postinfection, cell culture supernatants and whole-cell lysates were harvested and plaque assayed or analyzed by Western blotting.

## RESULTS

### Expression of the EGFP–CCHFV N fusion protein.

**FIG 1:**
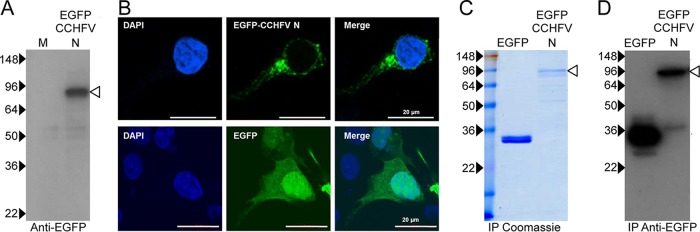
Expression and immunoprecipitation of EGFP–CCHFV-N in HEK293T cells. (A) HEK293T cells were transfected with a plasmid designed to express an EGFP–CCHFV-N fusion protein, which was detected by Western blot analysis of cell lysates using an anti-CCHFV N antibody. M, molecular mass marker lane (values at the left are in kilodaltons). (B) HEK293T cells were transfected with plasmids expressing EGFP or EGFP–CCHFV-N, and their subcellular localization was determined using confocal microscopy, with the nucleus stained blue using DAPI and with EGFP and EGFP–CCHFV-N detected as green. (C and D) Following GFP-trap-mediated IP from EGFP- and EGFP–CCHFV-N-expressing cells, proteins were resolved by SDS-PAGE and visualized by either Coomassie staining (C) or Western blotting (D) with an anti-GFP antibody. Unfilled arrowheads denote EGFP–CCHFV-N.

To identify cellular proteins that interacted with the nairovirus N protein during the replication cycle, an IP-based strategy was used to isolate the CCHFV N protein from cells in culture followed by mass spectrometry (MS) to identify coprecipitating cellular proteins. CCHFV N was initially expressed as an EGFP fusion protein (EGFP–CCHFV-N) in HEK293T cells, chosen for three principal reasons: first, their proven ability to support CCHFV replication; second, the availability of annotated human proteome databases to facilitate protein identification and function assignment; and third, their ability to be efficiently transfected using calcium phosphate. Western blot analysis of pEGFP-CCHFV-N-C2-transfected cells identified a protein with the expected 87-kDa molecular mass predicted for EGFP–CCHFV-N (Fig. 1A). Direct immunofluorescence (IF) confocal analysis of EGFP–CCHFV-N-expressing HEK293T cells identified fluorescence in perinuclear regions and also in discrete puncta elsewhere in the cytoplasm (Fig. 1B), a distribution that has been previously described for N during CCHFV infection (23, 31, 32) (see also Fig. 3C). This distribution contrasts with the localization of EGFP alone, expressed from pEGFP-C2, which was detected diffusely throughout both the cytoplasm and nucleus (Fig. 1B).

### Immunoprecipitation of EGFP–CCHFV-N.

To identify cellular proteins that interacted only with the N moiety of the EGFP–CCHFV-N fusion protein, the precipitated proteins from two different cell populations, one expressing EGFP–CCHFV-N and the other expressing EGFP alone, were compared. Comparison of the resulting data sets facilitated the elimination of likely nonspecific interaction partners and guided further experimentation to identify proteins specifically binding to CCHFV N.

EGFP–CCHFV-N and EGFP were isolated from separate cultures of HEK293T cells by IP using a protocol involving a so-called GFP-trap, which comprises a single-chain anti-EGFP monoclonal antibody conjugated to agarose. Clarified cell lysates were incubated with the GFP-trap to allow EGFP or EGFP–CCHFV N to bind, along with the associated cellular proteins, and then bound proteins were examined by SDS-PAGE followed by either Coomassie staining (Fig. 1C) or Western blotting (Fig. 1D) using an anti-EGFP and/or anti-CCHFV N antibody. Equal volumes of each eluate were mixed together, and combined IP samples were analyzed by mass spectrometry (MS).

To quantify subsequently detected CCHFV N binding proteins, stable isotope labeling by amino acids in cell culture (SILAC) was used in which the entire cellular proteome was metabolically labeled by incorporation of amino acids containing stable isotopes. Cells expressing EGFP were grown using medium media (containing isotopically labeled arginine and lysine), whereas cells expressing EGFP–CCHFV-N were grown in light media (without labeled amino acids). After the IP samples were mixed, distinct masses of precipitated peptides were resolved by MS, which revealed the identities and quantities of proteins associated with EGFP–CCHFV-N and of those associated with EGFP, allowing the relative protein quantities to be represented as a fold abundance difference value. Cell proteins associated with CCHFV-N would be expected to be highly abundant in the EGFP–CCHFV-N precipitated fraction and to be in low abundance (or absent) in the EGFP precipitated fraction and so would give a correspondingly high abundance ratio.

### Identification of cellular proteins that interact with the CCHFV N protein.

In order to provide increased confidence in our data sets, the LC-MS/MS analysis of cellular proteins that coprecipitated with EGFP–CCHFV N and EGFP was carried out twice. A total of 192 proteins were identified, with 112 common to both analyses, 46 proteins unique to the first analysis, and 34 unique to the second analysis, and all these are listed in Data set S1 in the supplemental material.

**FIG 2:**
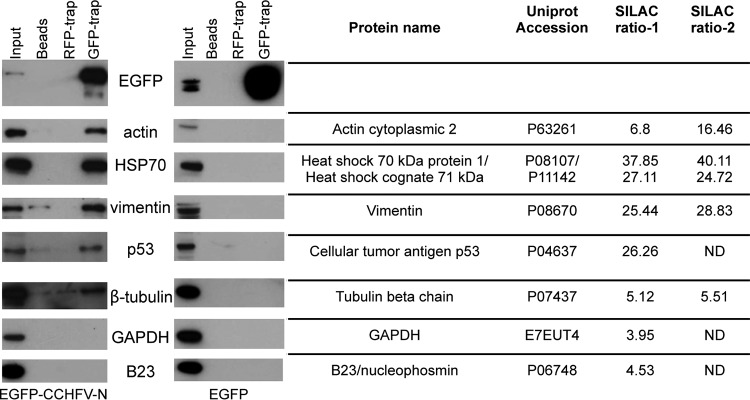
Validation of selected CCHFV N protein cellular interaction partners. HEK293T cells were transfected with EGFP–CCHFV-N and EGFP, and, 24 h later, cell lysate components were immunoprecipitated with GFP-trap, RFP-trap, or unconjugated agarose beads. The resulting IPs were separated by PAGE and then subjected to Western blotting to probe for the presence of the selected cellular proteins listed using specific antibodies. The ratios of the abundances of these proteins determined by the two SILAC LC-MS/MS analyses are indicated, and a complete list of all interaction partners is provided as an annotated table in Data set S1 in the supplemental material.

Previous studies have suggested that the majority of proteins identified by MS in SILAC-based GFP-trap experiments represent proteins that bind nonspecifically to EGFP or to the GFP-trap agarose matrix (33, 34). However, the ratio of the abundance of cellular proteins precipitated with EGFP–CCHFV-N to the abundance of those precipitated with EGFP can guide interaction specificity, and previous studies suggest that a ratio of 5 represents a conservative threshold under which interactions are likely nonspecific (34). A rational assessment of all identified proteins was performed in order to generate a priority listing of putative interactors; the basis for this selection process was a combination of the calculated EGFP–CCHFV-N/EGFP abundance ratio (approximately 5 or greater in both analyses) and a high significance value, calculated from the unique peptide counts and their corresponding abundances. The resulting 6 most likely authentic interacting proteins are listed in Fig. 2. As described above, the CCHFV N protein has previously been shown to bind actin (23), and so this known interaction represented an opportunity to assess the success of the GFP-trap and MS/MS identification procedure. In good agreement with this work, cytoplasmic actin was identified in both LC-MS/MS analyses by detection of 19 and 17 peptides with increased ratios of 6.8-fold and 16.5-fold, respectively.

### Independent validation of interacting partners by Western blot analysis.

As described above, a list of cellular proteins that potentially interacted with CCHFV N was derived from the MS analysis of IPs using EGFP–CCHFV-N protein. Next, IPs were repeated using unlabeled HEK293T cell lysates expressing either EGFP–CCHFV-N or native EGFP, in order to verify the putative protein-protein interactions. To determine if cell proteins were precipitated due to nonspecific interactions with components of the GFP-trap, control IPs were performed using both uncoupled agarose beads and a non-cross-reacting red fluorescent protein trap (RFP-trap) in which antibodies specific for RFP replaced those specific for GFP. As expected, Western blot analysis showed that the GFP-trap specifically immunoprecipitated EGFP–CCHFV-N and EGFP, whereas the RFP-trap and the agarose bead controls did not (Fig. 2, row 1), thus confirming the effectiveness of the GFP-trap for specific precipitation of EGFP and EGFP–CCHFV-N.

The nonlabeled IPs were probed by Western blotting using antibodies against selected cellular proteins identified by the LC-MS/MS analysis. As described above, cellular actin is known to bind CCHFV N protein (23), and so this known interaction was again used as a positive control. In agreement with the previous work, Western blot analysis of IPs using EGFP–CCHFV-N-transfected cell lysates revealed that actin was abundantly coimmunoprecipitated using the GFP-trap but that no actin was detected following IPs using the control RFP-trap or agarose beads alone (Fig. 2, row 2). Other cellular components that exhibited high EGFP–CCHFV-N/EGFP binding ratios and were identified by multiple peptides included heat shock protein 70 (HSP70), heat shock cognate 71-kDa protein (HSC70), vimentin, the p53 tumor suppressor protein, and tubulin. For each of these cellular proteins, Western blot analysis of IPs from EGFP–CCHFV-N-expressing cells detected a corresponding protein of the appropriate molecular mass both in the input material and following IP using the GFP-trap, with no binding or much reduced binding to either the agarose beads alone or the RFP-trap (Fig. 2, rows 3 to 6). None of these chosen cellular proteins were identified in similar quantities in GFP-trap IPs from cells expressing EGFP alone, indicating that the interaction between these cellular proteins is EGFP–CCHFV-N specific.

GAPDH and nucleophosmin (B23.1/NPM1) were identified in the LC-MS/MS analysis as having low abundance ratios in EGFP–CCHFV-N/EGFP IPs of 4 and 4.5, respectively, indicative of likely nonspecific interactions (34). In agreement with this, our Western blot analysis failed to detect the presence of either of these proteins in the IPs from the GFP trap when EGFP–CCHFV-N was expressed in cells (Fig. 2, rows 7 and 8). In addition, β-tubulin also exhibited similarly low ratios of 5.1 and 5.5 in the two LC-MS/MS analyses. Consistent with this, subsequent Western blot analysis detected β-tubulin in the GFP-trap IPs from EGFP–CCHFV-N-transfected cells with much reduced intensity compared to input cell lysate, suggesting that β-tubulin may represent a weak or transient interacting protein.

### Validation of the HSP70-N interaction using native CCHFV N.

**FIG 3:**
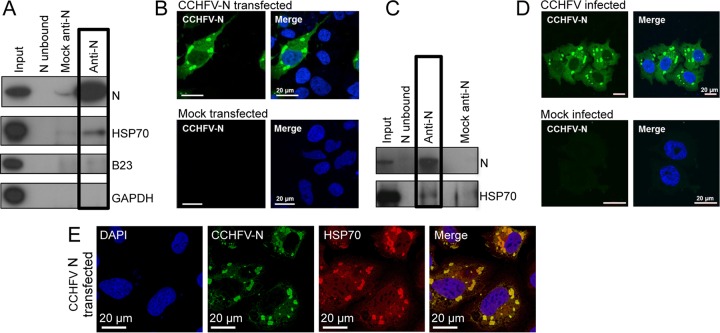
Interaction between cellular HSP70 and native CCHFV N expressed transiently or during CCHFV infection. (A) Anti-CCHFV N antisera was used for IP of native CCHFV N expressed in HEK293T cells, and the isolated proteins were analyzed by Western blotting using the antibodies shown. (B) The subcellular localization of native CCHFV N in HUH-7 cells was investigated using indirect immunofluorescence microscopy using anti-CCHFV N antisera, shown as green. Nuclei are stained with DAPI, shown as blue. (C) Anti-CCHFV N antisera was used for IP of native CCHFV N from CCHFV-infected SW13 cells, and the isolated proteins were analyzed by Western blotting using the antibodies shown. (D) The subcellular localization of native CCHFV N in CCHFV-infected SW13 cells was investigated by indirect immune fluorescence microscopy using anti-CCHFV N antisera, shown as green. The nuclei are stained with DAPI, shown as blue. (E) Indirect confocal immunofluorescence analysis of SW13 cells transiently expressing native CCHFV-N protein expressed from a plasmid cDNA. Cells were costained using antibodies specific for CCHFV N (green) and anti-HSP70 (red), with donkey and chicken secondary antibodies, respectively, as well as DAPI (blue).

The results described in the previous section identified cellular proteins that interacted with the EGFP CCHFV N fusion protein, including HSP70 and HSC70, which are members of a group of related proteins that form the HSP70 family of cellular chaperones (35). Functional interactions between heat shock proteins and viral components have recently been documented in other RNA viruses, and so we further pursued this potential interaction. In order to exclude the possibility that the association between HSP70 and N was influenced by the EGFP moiety, native CCHFV N protein was expressed both transiently in HEK293T cells and during authentic CCHFV infection, followed by its precipitation with anti-CCHFV N antibodies made in-house. The specificity of these antisera was confirmed by Western blot analysis using material harvested from HEK293T cells transfected with a CCHFV N expression plasmid as well as lysates from CCHFV-infected SW13 cells. In both cases, the CCHFV N antisera detected a single band of the expected 54-kDa molecular mass (Fig. 3A and C, top rows, Input). The subcellular distributions of native CCHFV N expressed either transiently (Fig. 3B) or in CCHFV-infected SW13 cells (Fig. 3D) were indistinguishable, being predominantly perinuclear, with additional puncta containing CCHFV N detected in the cytoplasm.

IP of native CCHFV N from both infected and transfected cells was achieved using Dynabeads conjugated to anti-CCHFV N antibodies, after which precipitated proteins were analyzed by Western blotting. The effectiveness of the IP procedure was confirmed by the detection of abundant CCHFV N protein in IP samples (Fig. 3A and C, top rows, anti-CCHFV N) and the absence of CCHFV N in the lanes corresponding to mock-transfected or mock-infected cell lysates (Fig. 3A and B, top row, Mock anti-N) and unconjugated antibodies incubated with transfected or infected cell lysates (Fig. 3A and B, top row, N unbound). The IP samples were analyzed by Western blotting using an anti-HSP70 antibody, which revealed the abundant presence of a corresponding 70-kDa protein in the anti-CCHFV N IP samples from transiently transfected cells, with no or little protein detected in mock-transfected cells (Fig. 3A, boxed lanes). Similarly, anti-HSP70 Western blot analysis of N protein precipitated from CCHFV-infected cells identified a 70-kDa protein, with little or no corresponding protein detected in mock-infected cells (Fig. 3B, boxed lanes). The two cellular proteins that were not considered to be CCHFV N interacting partners, GAPDH and B23.1 (nucleophosmin), were chosen to act as negative controls. Consistent with the proteomics analysis, neither of these cellular proteins was detected by Western blotting in the anti-CCHFV N IP samples.

To further examine the interaction between CCHFV N and HSP70/HSC70, indirect IF confocal microscopy was performed to determine the cellular localization of endogenous HSP70 and native CCHFV N transiently expressed in SW13 cells. This analysis showed that HSP70/HSC70 was present at multiple discrete locations throughout the cell, with staining present predominantly within the cytoplasm, and often with dense staining in perinuclear regions of both transfected and untransfected cells (Fig. 3E). Costaining of cells with CCHFV N antisera revealed a predominantly perinuclear distribution, and, in many instances, the CCHFV N-positive puncta colocalized with HSP70/HSC70. This suggested that a subset of HSP70/HSC70 molecules were associated with CCHFV N, although abundant unassociated HSP70/HSC70 was also detected within infected cells, which was expected due to its native chaperone roles.

### HAZV N protein interacts with HSP70 inside infected cells.

The results described in the previous section demonstrated that HSP70 interacted with CCHFV N in infected cells. To determine whether this interaction was a common feature of nairovirus N proteins, this interaction was also investigated using HAZV N, exploiting the significant logistical advantages of working under BSL2 conditions rather than under the highly restrictive maximum containment conditions required for CCHFV.

**FIG 4:**
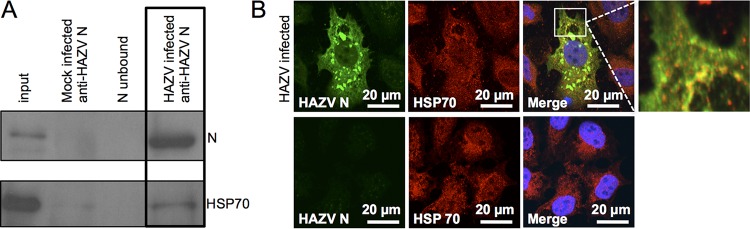
Analysis of the interaction between HAZV N and HSP70 in cells. (A) HAZV N was immunoprecipitated from HAZV-infected cells using anti-N antisera and then subjected to Western blotting using the antibodies shown. (B) Indirect confocal immunofluorescence analysis of SW13 cells infected with HAZV revealed HAZV N (green) localized in discrete cytoplasmic puncta, similarly to CCHFV N. HSP70 (red) was present in both the cytoplasm and nucleus and also colocalized with HAZV N in some cytoplasmic puncta. Nuclei are stained with DAPI, shown as blue. Bottom row shows mock-infected cells.

SW13 cells were infected with HAZV at an MOI of 1, and N protein was precipitated 24 h after infection by the use of anti-HAZV N antisera under the same conditions used to IP native CCHFV N. The ability of the anti-HAZV N antiserum to detect HAZV N was confirmed by Western blot analysis of cell lysates infected with HAZV, which detected a band of the appropriate molecular mass (54 kDa); HAZV N was detected in the input and the anti-HAZV N IP lanes but was absent from the negative-control lanes. Next, the co-IP of HSP70/HSC70 with HAZV N was confirmed by Western blot analysis of IP samples using an anti-HSP70 antibody. This specifically identified a 70-kDa protein in the input sample and also in the anti-HAZV N IP sample (Fig. 4A), thus identifying HSP70/HSC70 as an HAZV N interaction partner within infected cells. HSP70/HSC70 was not detected or was detected to a much lesser extent in the IP control lanes from mock-infected cells or in precipitations performed using the unconjugated Dynabead matrix.

To further evaluate the interaction between N and HSP70 during nairovirus infection, indirect IF confocal microscopy was used to determine the subcellular localization of both N and HSP70/HSC70 during HAZV infection. As described above for CCHFV N, indirect IF analysis showed that HSP70/HSC70 partially colocalized with HAZV N in sites that were predominantly perinuclear, but staining of HSP70/HSC70 in sites that were free of HAZV N was also evident (Fig. 4B). Taken together, the results suggest that a subset of HSP70/HSC70 molecules are associated with HAZV N during infection but that free HSP70/HSC70 also exists within infected cells, presumably performing its native chaperone function with other client proteins.

### Identification of host cell proteins packaged within nairovirus particles.

**FIG 5:**
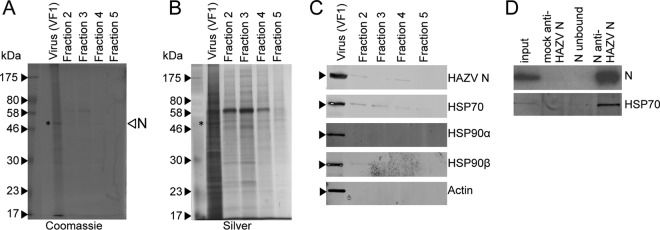
SDS-PAGE analysis of purified HAZV harvested from SW13 cell supernatant and Western blot confirmation of the presence of HSP70 and HSP90 family members. (A and B) Secreted HAZV particles harvested from infected SW13 cell supernatants were purified by PEG precipitation followed by centrifugation through an iodixanol gradient. HAZV particles were isolated (lane VF1), and the remaining 4 fractions (1 ml each) were collected from the top of the gradient (Fraction 2) to the bottom of the gradient (Fraction 5). Fractions were analyzed by SDS-PAGE and either Coomassie stained (A) or silver stained (B). (C) Fraction VF1 was subjected to Western blot analysis using both anti-HAZV N antisera and antibodies targeting the listed cellular proteins, including HSP70. (D) HAZV N was immunoprecipitated from purified infectious HAZV and then subjected to Western blot analysis using anti-HAZV N and anti-HSP70 antibodies.

The experiments described above showed that the nairovirus N protein interacts with members of the HSP70 family within virus-infected cells. One hypothesis that follows from this is that HSP70 influences the conformation of RNPs during their assembly into progeny virions, which would predict that the chaperone would be present within assembled and budded virus. To test this, the composition of infectious nairovirus particles that are released from cells was investigated. As maximum containment protocols are required to grow and purify CCHFV, which is problematical, HAZV was used for this analysis instead. HAZV released from cells was purified by ultracentrifugation on an iodixanol gradient, resulting in a narrow opalescent band that was aspirated (fraction VF1), with the remaining contents of the gradient being collected as 4 sequential fractions (F2 to F5). All five fractions were analyzed by SDS-PAGE followed by Coomassie staining (Fig. 5A) or silver staining (Fig. 5B), which showed a sharp peak in protein abundance in the initially aspirated VF1 fraction (the protein corresponding to the molecular mass of N [shown by an asterisk]). Higher-molecular-mass proteins were visible which may correspond to virus components Gn/Gc and L, as well as proteins that were likely cellular in origin (Fig. 5A and B). All fractions were analyzed for the presence of HAZV N protein by Western blotting using anti-HAZV N antibody (Fig. 5C, top row), which revealed that HAZV N protein was almost exclusively found in fraction VF1. Further confirmation that VF1 contained purified HAZV was that only VF1 was demonstrated to possess infectious virus by plaque assay, having a titer of 2 × 10^5^ PFU/ml.

In order to elucidate the protein composition of purified infectious HAZV, fraction VF1 was denatured and separated by SDS-PAGE and proteins were identified by MS. In addition, the content of fraction F3 was also analyzed by MS to identify proteins present throughout the gradient as background contaminants. A total of 264 proteins were identified in VF1, and 82 proteins were identified in F3 (see Data Set S2 in the supplemental material). Of these 82 proteins, 69 were also identified in VF1. Unless the abundances of these proteins were considerably increased within VF1, they were likely dispersed throughout the gradient rather than being associated within intact HAZV particles and thus could be discounted from further analysis. The peptides identified in fractions VF1 and F3 were ranked in accordance with their calculated exponentially modified protein abundance index (emPAI) score. As expected, the HAZV N, Gn, Gc, and L proteins were detected in VF1, as was actin, previously reported to interact with the nairovirus N protein within cells (23). In contrast, analysis of fraction F3 showed that actin and all HAZV proteins were either absent or present in negligible quantities.

The MS analysis of VF1 identified peptides corresponding to several members of the HSP70 family and also to members of the HSP90 family. These findings were validated by performing Western blot analysis on VF1, using antibodies specific for HSP70 and HSP90 family members, which revealed the abundant presence of proteins corresponding to HSP70/HSC70 and HSP90-alpha and HSP90-beta (Fig. 5C). Trace quantities of these chaperones were also detected in other fractions but in vastly reduced abundance compared to VF1, consistent with their being highly enriched in purified virus particles.

### HSP70/HSC70 associated with HAZV N protein in infectious particles.

The findings described in the previous section indicated the abundant presence of cellular chaperones of the HSP70 family within purified HAZV particles. Next, we investigated whether these chaperones were present within the HAZV particles in association with HAZV N by subjecting VF1 to IP using anti-HAZV N antisera. The subsequently precipitated proteins were separated by SDS-PAGE and then probed by Western blot analysis using anti-HSP70 antibodies, which detected a 70-kDa protein consistent with the HSP70/HSC70 targets. The lack of a similar identified protein in the control lanes from IPs performed using the unconjugated Dynabead matrix or from F3 confirmed the specificity of this interaction (Fig. 5D). Taken together, these results suggest that incorporation of HSP70/HSC70 in infectious HAZV particles was mediated through specific interaction with N, most likely in the form of RNPs.

### Inhibition of HSP70 function using small-molecule inhibitors.

To further examine the importance of HSP70 family chaperones during the nairovirus replication cycle, the effects on HAZV replication of two previously characterized HSP70 inhibitors, pifithrin-μ (PIF) and VER155008 (VER), were evaluated. HSP70 and its homologs comprise two functionally distinct domains, namely, an N-terminal substrate-binding domain (SBD) and a C-terminal ATPase domain that allosterically modulates the activity of the SBD (35). PIF directly interacts with the SBD, thus interfering with its ability to bind to client proteins, whereas VER targets the ATPase domain, preventing ATP from binding and consequently inhibiting HSP70 function by preventing allosteric regulation of the SBD.

**FIG 6:**
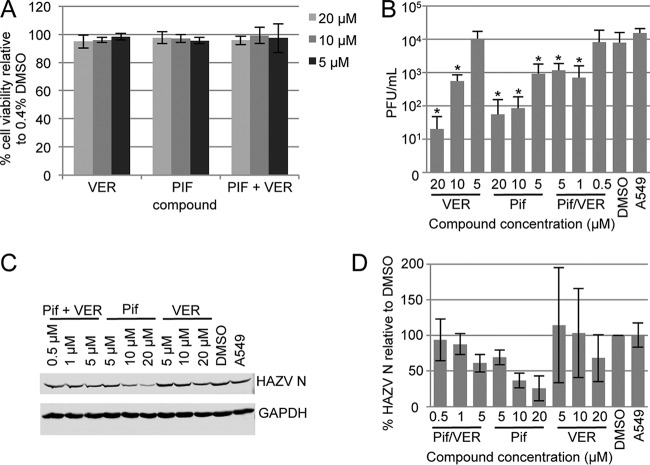
The effect of HSP70/HSC70 inhibitors VER and PIF on HAZV replication. (A) Cytotoxicity of VER and PIF in SW13 cells was assessed by MTT assay after 24 h of incubation of SW13 cells with VER and PIF at several different concentrations in the absence of HAZV. Cytotoxicity levels are presented relative to 0.4% DMSO results. (B) The effect on HAZV replication of treating A549 cells with VER and PIF was determined by plaque assay, which revealed a reduction in HAZV titers at subcytotoxic PIF and VER concentrations. The statistical significance of decreased virus multiplication using PIF and VER in relation to DMSO-only controls was assessed using paired Student's *t* tests, and significance is shown with an asterisk (* [*P* < 0.05]). (C) Western blot analysis of HAZV N protein and GAPDH levels within infected cells following PIF and VER treatment at subcytotoxic concentrations. (D) HAZV N protein levels quantified from Western blot analyses performed in three experiments.

As described above, HAZV was utilized for this study due to the relative ease of handling HAZV compared to CCHFV. In order to assess the effects of VER and PIF on HAZV multiplication, we first established their cytotoxicity profiles in A549 cells by MTT [3-(4,5-dimethyl-2-thiazolyl)-2,5-diphenyl-2Htetrazolium bromide] assay (Fig. 6A) in comparison to a baseline of 0.4% DMSO, which exceeded the maximal concentration of the solvent used to deliver the compounds. Based on previous analyses of HSP70 inhibition by our group (36), inhibitor concentrations of between 5 and 20 μM were tested, which resulted in no detectable cellular toxicity and no reduction in GAPDH expression. We next tested the ability of VER and PIF at 5, 10, and 20 μM concentrations to interfere with virus replication, as determined by plaque assay. As the plaque assay measures the yield of secreted infectious virus, it would determine how PIF and VER influence all intracellular stages of the virus replication cycle as well as the ability of released virus to infect new cells.

Incubation of HAZV-infected A549 cells with 20 μM VER for 24 h reduced HAZV titers by approximately 1,000-fold compared to the DMSO control results, whereas over the same 24-h period, incubation with 20 μM PIF reduced virus titers by approximately 500-fold (Fig. 6B). This indicated that the production of infectious HAZV virions was greatly diminished in the presence of these compounds. These findings establish HSP70/HSC70 as having a critical role in the nairovirus replication cycle.

To better understand this role, we next wanted to gain information on the specific stage of the virus replication cycle at which HSP70 and HSC70 acted. To achieve this aim, we examined the effect of PIF and VER on the quantity of HAZV N protein that was expressed during infection (Fig. 6C and D). This analysis revealed a gradual decrease in HAZV N expression as the concentration of VER increased, such that N expression was approximately 70% that of the DMSO control at a subtoxic VER concentration of 20 μM. When cells were incubated with PIF, the use of the same subtoxic 20 μM concentration resulted in a greater decrease in N expression levels to approximately 25% of that seen with the DMSO controls (Fig. 6C and D). These findings suggest that HSP70/HSC70 is required during the HAZV replication cycle at a stage prior to protein synthesis, such as genome replication or gene expression, rather than at a later stage, such as virus assembly and egress.

## DISCUSSION

RNA viruses have limited coding capacity; to compensate for this, many of their proteins exhibit remarkable structural economy, often possessing multiple functions. An additional strategy that viruses have adopted to increase their functional repertoire is that of subverting the function of host cell components. Based on the hypothesis that nairovirus N proteins must interact with specific host cell proteins to complete the infectious cycle, we aimed to determine the identity of these associating cellular factors. To achieve this aim, we utilized both quantitative proteomics and conventional IP protocols to elucidate the identity of interacting cellular proteins that coprecipitate with CCHFV and HAZV N proteins during both the intracellular and extracellular phases of their replication cycles.

Our results indicated that many interacting proteins of CCHFV N were components of the cellular cytoskeleton, including actin, tubulin, and vimentin. Relevant to the current study, CCHFV N has previously been shown to interact with actin, and this provides further confidence in our findings. The role of these cytoskeletal components in the CCHFV replication cycle is unclear, although strong possibilities must include the trafficking of viral components from the site of virus entry or uncoating to the virus factories where synthesis of viral components for later assembly of progeny virions takes place. However, the most abundant interacting partners of CCHFV N were members of the HSP70 family of ATP-dependent cellular chaperones, which, in association with DnaJ cofactor adapter proteins, perform roles that relate to correct folding and transport of newly synthesized and misfolded proteins, as well as to the assembly of multicomponent complexes (37). We also showed that the N protein of the closely related HAZV also associates with HSP70 family members, not only within infected cells, but also in secreted infectious virus particles. Due to the extremely close structural similarities of these two N proteins (14), we suggest that the N-HSP70 interactions that we have observed in both HAZV and CCHFV are similarly related. This reasoning led us to test the functional role of HSP70 family members throughout the HAZV replication cycle and to propose that the findings of this analysis would also apply to the infectious cycle of CCHFV.

Growing evidence from the literature indicates that members of the HSP70 family of chaperones functionally interact with proteins belonging to viruses classified in many different groups spanning the DNA and RNA viruses infecting a bewildering array of unicellular and multicellular organisms. For negative-stranded RNA viruses (of which nairoviruses are examples), the most commonly reported role of HSP70/HSC70 is its interaction with components of the viral RNA synthesis machinery, which influences both transcription and replication of viral RNA. Examples include HSP70/HSC70 interactions with the N proteins of rabies virus (38), measles virus (39), and Hantaan virus (40), polymerase subunits PB1 and PB2 from influenza A virus (FLUAV) (41, 42), and components of the human respiratory syncytial virus (HRSV) polymerase complex (36, 43–45). Interestingly, the role of HSP70/HSC70 in RNA synthetic processes has been shown to be sometimes stimulatory and sometimes inhibitory. In the case of HRSV, depletion or inhibition of the associated heat shock proteins was found to have an inhibitory effect on RNA synthesis, suggesting that HSP70/HSC70 had a positive role in the viral replication cycle, and it was postulated that the chaperone enhanced polymerase activity by facilitating its correct folding. In contrast, the role of HSP70 in FLUAV RNA synthesis is apparently more complex, with initial reports suggesting that it disrupts polymerase-RNA binding, leading to reduced transcription and replication (41). However, more-recent conflicting reports indicate that FLUAV RNA synthesis is stimulated by physiological concentrations of HSP70 but inhibited when HSP70 is overexpressed through heat shock stimulation or transient expression (42).

In order to assess whether the HSP70/HSC70 interaction was beneficial or detrimental to the nairovirus replication cycle, we tested the impact on HAZV replication of modulating HSP70/HSC70 function using the previously described HSP70/HSC70 inhibitors PIF and VER (46). These chaperones, along with their cofactors, are emerging as potent and promising antiviral targets (47). PIF and VER were carefully titrated to ensure that the concentrations used were noncytotoxic as measured by MTT assay and expression of cellular GAPDH. For both inhibitors, the yield in secreted infectious virus was dramatically reduced by at least 90% when used at 20 μM, a concentration which was conservatively chosen to rule out any off-target effects. Taken together, these consistent findings suggest that HSP70/HSC70 plays an important beneficial role in the HAZV replication cycle that is independent of the native role of HSP70/HSC70 in host cell processes.

An important question that we now wish to ask is this: at what stage of the nairovirus replication cycle is HSP70/HSC70 acting, such that it is essential for virus production? The finding that PIF and VER both cause a dramatic reduction in the accumulation of N protein at nontoxic concentrations suggests that HSC70/HSP70 likely influences one or more early stages of the replication cycle prior to, or including, protein production, such as virus entry, uncoating, RNA synthesis, or protein synthesis. The packaging of these chaperones in association with RNPs within a secreted, infectious particle is also consistent with an early role in the viral replication cycle, when RNPs are released from the virion into the cytosol. Possible roles include facilitating RNP conformational changes that may be required for dissociation of RNPs from the glycoprotein endodomains and decondensation of RNPs in preparation for primary transcription. Interestingly, a role for HSP70/HSC70 in early RNP functions has been proposed for canine distemper virus (48), another negative-stranded RNA virus. Those authors suggested that HSP70/HSC70 could be required for remodeling of the RNP template, perhaps from a condensed to a more relaxed conformation, and that this switch may be needed for gene expression. However, HSP70 family members and their associated DnaJ cofactors may play important proviral roles in more than one stage of the nairovirus life cycle. Dependence on the HSP70/DnaJ chaperone network at all stages of the dengue virus 2 (DENV-2) replication cycle was recently comprehensively dissected, assigning defined roles to multiple HSP70 isoforms and DnaJ protein combinations (47). That work revealed an important role for cytosolic cofactor DnaJ A2 during DENV RNA replication, and, interestingly, this factor was detected in the MS analysis of precipitated CCHFV N, also consistent with a role in nairovirus RNA replication. It is also noteworthy that the DnaJ B11 cofactor found to be required for DENV assembly was the sole DnaJ cofactor found in HAZV particles, also suggestive of the possibility that members of the HSP70/DnaJ network represent proviral cell factors that are required across diverse virus families.

In regard to the role of the N-HSP70 interaction, it has previously been shown that heat shock proteins rescue the ability of mutant proteins to adopt a native conformation (49). In the context of RNA viruses, this ability may be particularly beneficial with respect to mitigating the consequences of their high genome mutation rates, which could otherwise result in the generation of pools of misfolded viral proteins. It has been suggested that large multisubunit complexes that depend on the precise geometry of interacting components may be particularly vulnerable to protein misfolding, as a single misfolded subunit may render the entire complex defective (50). In the case of the negative-stranded RNA viruses such as nairoviruses, they possess RNP complexes built from multiple copies of N protein in association with the RNA genome, as well as an associated polymerase. The nairovirus L segment is over 12,000 nucleotides long and is likely encapsidated by over 1,000 N protein monomers that enwrap the long L segment RNA. It is feasible that the nairoviruses may have developed a strategy involving cellular chaperones to protect this significant metabolic investment in the face of frequent polymerase error. A better understanding of critical virus-host cell interactions such as the N-HSP70 interaction may aid in the design of effective strategies for control of pathogenic viruses within the Nairovirus genus, as well as of other related viruses. This may be particularly promising in the case of heat shock proteins, as many of the corresponding inhibitory compounds are well characterized and as viruses appear to be unable to develop resistance (51).

## Supplementary Material

Supplemental material
