# Supplementary material for: Heat Shock Protein 70 Family Members Interact with Crimean-Congo Hemorrhagic Fever Virus and Hazara Virus Nucleocapsid Proteins and Perform a Functional Role in the Nairovirus Replication Cycle
Source: J Virol. 2016 Sep 29;90(20):9305–16. doi: 10.1128/JVI.00661-16 (PMC5044845; doi:10.1128/JVI.00661-16)
Supplement: Supplemental material [file supp_90_20_9305__index.html]

Heat Shock Protein 70 Family Members Interact with Crimean-Congo Hemorrhagic Fever Virus and Hazara Virus Nucleocapsid Proteins and Perform a Functional Role in the Nairovirus Replication Cycle — Supplemental material 

# Heat Shock Protein 70 Family Members Interact with Crimean-Congo Hemorrhagic Fever Virus and Hazara Virus Nucleocapsid Proteins and Perform a Functional Role in the Nairovirus Replication Cycle

## Supplemental material

- Supplemental file 1 -

  Data set S1 (Proteomics data set for CCHFV N protein IP.)

  XLS, 136K
- Supplemental file 2 -

  Data set 2 (Proteomics data set for purified HAZV.)

  XLSX, 163K
